# Effect of Accelerator in Green Synthesis of Silver Nanoparticles

**DOI:** 10.3390/ijms11103898

**Published:** 2010-10-12

**Authors:** Majid Darroudi, Mansor Bin Ahmad, Abdul Halim Abdullah, Nor Azowa Ibrahim, Kamyar Shameli

**Affiliations:** 1 Advanced Materials and Nanotechnology Laboratory, Institute of Advanced Technology (ITMA), Universiti Putra Malaysia, 43400 UPM Serdang, Selangor, Malaysia; E-Mail: majiddarroudi@gmail.com; 2 Department of Chemistry, Universiti Putra Malaysia, 43400 UPM Serdang, Selangor, Malaysia; E-Mails: halim@science.upm.edu.my (A.H.A.); norazowa@science.upm.edu.my (N.A.I.); kamyarshameli@gmail.com (K.S.)

**Keywords:** silver nanoparticles, green synthesis, stabilizer, gelatin, glucose, accelerator

## Abstract

Silver nanoparticles (Ag-NPs) were successfully synthesized in the natural polymeric matrix. Silver nitrate, gelatin, glucose, and sodium hydroxide have been used as silver precursor, stabilizer, reducing agent, and accelerator reagent, respectively. This study investigated the role of NaOH as the accelerator. The resultant products have been confirmed to be Ag-NPs using powder X-ray diffraction (PXRD), UV-vis spectroscopy, and transmission electron microscopy (TEM). The colloidal sols of Ag-NPs obtained at different volumes of NaOH show strong and different surface plasmon resonance (SPR) peaks, which can be explained from the TEM images of Ag-NPs and their particle size distribution. Compared with other synthetic methods, this work is green, rapid, and simple to use. The newly prepared Ag-NPs may have many potential applications in chemical and biological industries.

## 1. Introduction

Nowadays, nanomaterials have been improved as a result of truly important recent advances by applying “green” chemistry rules to nanotechnology and materials science. Silver nanoparticles (Ag-NPs) have been widely used during the past few years in various applications, such as biomedicine [1,2], biosensor [3], and catalysis [4]. Many methods can be used to synthesize Ag-NPs, including microemulsion [5], γ-Ray [6], UV-irradiation [7], microwave irradiation [8], and spray pyrolysis [9]. Polymers have also been used as matrices in nanocomposites or as stabilizers to provide stability for the metal nanoparticles against oxidation, agglomeration, and precipitation [10,11]. Preparations of Ag-NPs in various polymers have been published to give well-dispersed Ag-NPs, such as polyvinylpyrrolidine (PVP) [12], polyethylene glycol [13], poly(methyl methacrylate) [14], poly (vinyl alcohol) [15], polyaniline [16], and polyacrylonitrile (PAN) [17]. Natural polymers have also been used because they are non-toxic and biocompatible. For example, natural rubber [18], polysaccharides [19], cellulose [20], chitosan [21], and starch [22] have been used as matrices or stabilizers for preparation of metallic nanoparticles. The present work is on the use of polymers as the stabilizing agent and use of OH as an accelerator in the synthesis of metallic nanoparticles e.g., Ag [23,24] and Cu [25]. Gelatin is a natural biopolymer extracted from the partial hydrolysis of collagen and has good biocompatibility and biodegradability; in recent years, it has been widely used in wound dressings, drug carriers, and tissue scaffolds [26].

The current work describes a green, rapid, and easy method for preparing well-dispersed Ag-NPs in natural polymeric media like gelatin. The morphologies of Ag-NPs and the mechanism of their formation by oxidation of glucose using an accelerator reagent are discussed.

## 2. Results and Discussion

**Figure 1:**
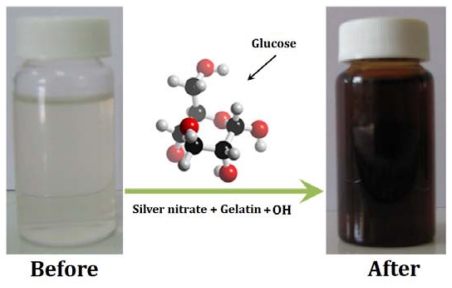
Schematic of the preparation process of Ag-NPs.

Glucose as an aldehyde can reduce silver ions to metallic silver and through this process oxidizes itself to glucolic acid. The obtained colloidal Ag-NPs were characterized by spectroscopic results. The formation of Ag-NPs was studied using UV-vis spectroscopy, which has proven to be a very useful technique for the analysis of nanoparticle formation over time. The color of the obtained samples at different volumes of NaOH rapidly changed from colorless to dark brown, indicating the formation of Ag-NPs (Figure 1).

**Figure 2:**
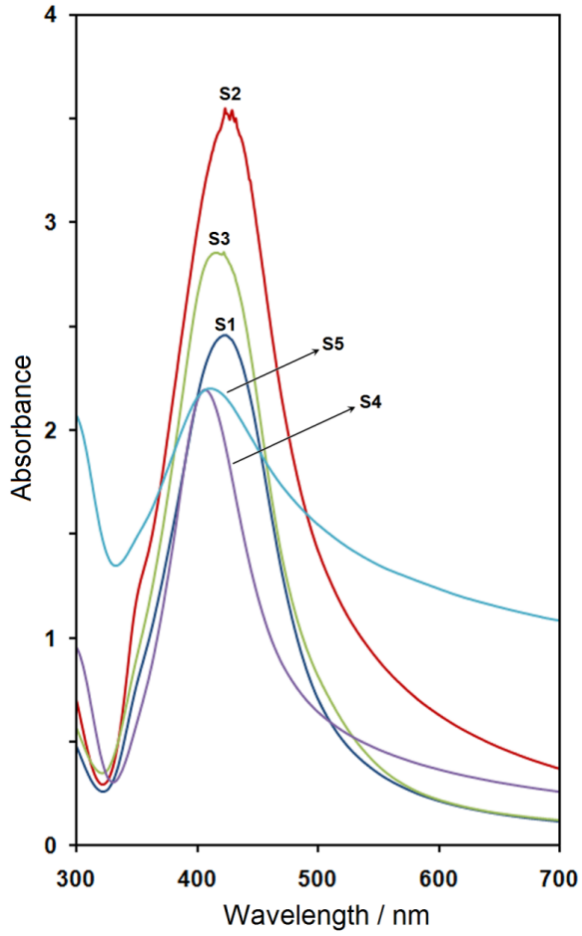
The UV-visible spectra of Ag-NPs prepared at different volumes of NaOH.

The UV-vis absorption spectra of prepared samples are shown in Figure 2. The obtained Ag-NPs displayed absorption peaks, the characteristic surface plasmon resonance (SPR) band for silver, centered from 407 to 424 nm. The location (λ_max_) of the SPR peaks and volumes of NaOH with corresponding pH are also summarized in Table 1.

As indicated, the first addition of NaOH led to the broadening of the SPR peak relative to the sample S1. Greater volumes of NaOH (S2) increased the absorbance due to increases in silver concentration [27]. In sample S3, this resulted in a stronger blue-shift in λ_max_ to 422 nm. In sample S4, the increased volume of accelerator led to the SPR peak being considerably blue-shifted to a lower wavelength (407 nm). This blue-shift is related toa decrease in the particle size of Ag-NPs [28] because the SPR band in metal nanoparticles displays the blue-shift based on the decreased size of particles. For S5, the λ_max_ was red-shifted to 412 nm due to the increased size of Ag-NPs [27,29]. These results demonstrated good agreement with the results obtained in the transmission electron miscroscopy (TEM) images of Ag-NPs and their particle size distribution.

**Figure 3:**
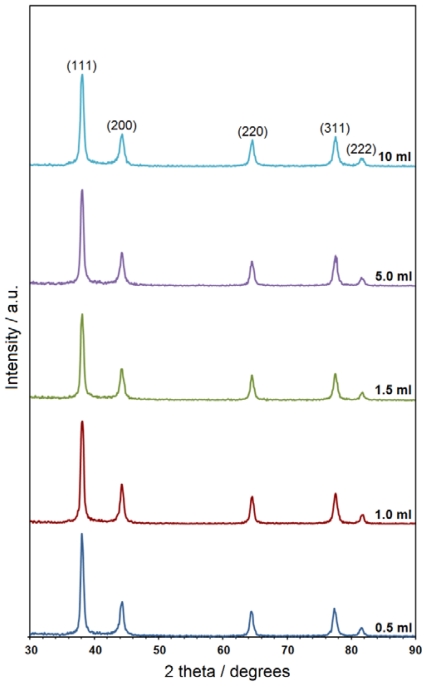
The PXRD patterns of Ag-NPs prepared at different volumes of NaOH.

The powder X-ray diffraction (PXRD) patterns of the prepared samples at different volumes of NaOH indicate the formation of the Ag-NPs. As shown in Figure 3, all samples have similar diffraction profiles. The PXRD peaks at 2θ of 35.9°, 42.1°, 62.8°, 75.7° and 82.7° can be attributed to the (111), (200), (220), (311) and (222) crystallographic planes of face-centered cubic (fcc) silver crystals, respectively (Ref. # 01-087-0597). For the all samples, the main crystalline phase was silver; no obvious impurities (e.g., Ag_2_O [22]) were evident in the PXRD patterns. Moreover, no other peaks related to an impurity, indicating that the silver metal was relatively pure. Gelatin was used as the stabilizer and matrix during this synthesis. The possible chemical equation for preparing the Ag-NPs is:

(1) $${\text{Ag}}_{\left(\text{aq}\right)}^{+}+{\text{gel}}_{\left(\text{aq}\right)}\to {\left[\text{Ag}(\text{gel})\right]}_{\left(\text{aq}\right)}^{+}$$

(2) $$2{\left[\text{Ag}(\text{gel})\right]}_{\left(\text{aq}\right)}^{+}+2{\text{OH}}^{-}+{\text{C}}_{5}{\text{H}}_{11}{\text{O}}_{5}-\text{CHO}\to 2\text{Ag}+2\text{gel}+{\text{H}}_{2}\text{O}+{\text{C}}_{5}{\text{H}}_{11}{\text{O}}_{5}-\text{COOH}$$

After dispersion of silver ions in the gelatin matrix (Equation 1), gelatin reacted with the Ag^+^ to form a stable gelatinous complex [Ag(gel)]^+^, which reacted with OH^−^ to form silver metal due to the reduction of silver ions through the oxidation of glucose to gluconic acid (Equation 2).

**Figure 4:**
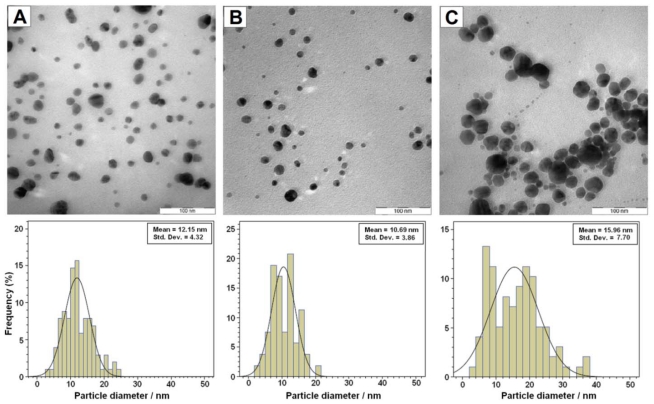
TEM images of Ag-NPs and their particle size distribution at different volumes of NaOH; 1.5 (**A**), 5 (**B**), and 10 mL (**C**).

The TEM images demonstrate the formation of Ag-NPs at different volumes of NaOH. Figure 4 shows typical TEM images and the corresponding particle size distribution of the prepared Ag-NPs. The TEM results indicated that the obtained samples retained a narrow particle size distribution and the average size of all prepared Ag-NPs was less than 20 nm, and a smaller size (~10.7 nm) was obtained for S4.

## 3. Experimental Section

### 3.1. Materials

All chemicals and reagents in this work were of analytical grade and used without any purification. Materials used for the synthesis of Ag-NPs included silver nitrate (Alfa-Aesar, 99.98%), gelatin (Sigma, Type A), D-glucose (BDH-Limited Poole, Analar), and sodium hydroxide (R & M Chemicals, 99.0%). Doubly distilled water was used throughout the experiments.

### 3.2. Synthesis of Silver Nanoparticles

In the present study, 1% wt. gelatin was added to an aqueous solution of AgNO_3_ (10 mL, 1 M) in a flask. Different volumes of aqueous solution of NaOH (1 M) were added to prepare various samples (0.5 (S1), 1.0 (S2), 1.5 (S3), 5 (S4) and 10 mL (S5)). These solutions were heated at 60 °C and then 10 mL of aqueous solution of glucose (2 M) was added. The suspension immediately turned brown in all samples, indicating the formation of silver nanoparticles. The reaction was continued for 15 minutes. The obtained suspensions were centrifuged at 15,000 rpm.

### 3.3. Characterization Methods and Instruments

The prepared Ag-NPs were characterized using ultraviolet–visible (UV-vis) spectroscopy, transmission electron microscopy (TEM) and powder X-ray diffraction (PXRD). The UV-vis spectra were recorded over the range of 300–700 nm with a Lambda 35-Perkin Elmer UV-vis spectrophotometer. TEM observations were conducted using a Hitachi H-7100 electron microscope while the particle size distribution were determined using the UTHSCSA Image Tool version 3.00 program. The structure of produced Ag-NPs has been studied using powder X-ray diffraction (PXRD, Philips, X’pert, Cu Kα). PXRD patterns were recorded at a scan speed of 2°/min. After reactions, the samples were centrifuged using a high-speed centrifuge machine (Avanti J25, Beckman).

## 4. Conclusions

We have demonstrated a green synthesis of high purity Ag-NPs with glucose reduction of aqueous AgNO_3_ at different volumes of NaOH as a reaction accelerator. Nanoparticle agglomeration was controlled with the addition of gelatin as a stabilizing agent. PXRD and TEM measurements displayed that the resultant nanoparticles were faced centered cubic (fcc) structures smaller than 20 nm in diameter. Thus, this work provides important advantages namely, simplicity, speed, and eco-friendliness.

## Figures and Tables

**Table 1:** The characteristics of Ag-NPs prepared at different volumes of NaOH.

Sample	Volume of NaOH (mL)	pH	λ_max_
S1	0.5	3.5	424
S2	1	3.3	423
S3	1.5	3.2	422
S4	5	3.7	407
S5	10	4.3	412

## References

[1] Schultz DA Plasmon resonant particles for biological detection Curr. Opin. Biotech 2003 14 13 22 12565997 10.1016/s0958-1669(02)00015-0

[2] Parak WJ Gerion D Pellegrino T Zanchet D Micheel C Williams SC Boudreau R Le Gros MA Larabell CA Alivisatos AP Biological applications of colloidal nanocrystals Nanotechnology 2003 14 15 27

[3] Nam JM Thaxton CS Mirkin CA Nanoparticle-based bio-bar codes for the ultrasensitive detection of proteins Science 2003 301 1884 1886 14512622 10.1126/science.1088755

[4] Haneda M Kintaichi Y Inaba M Hamada H Infrared study of catalytic reduction of nitrogen monoxide by propene over Ag/TiO_2_-ZrO_2_ Catal. Today 1998 42 127 135

[5] Husein MM Rodil E Vera JH A novel method for the preparation of silver chloride nanoparticles starting from their solid powder using microemulsions J. Colloid Interf. Sci 2005 288 457 467 10.1016/j.jcis.2005.03.023 15927613

[6] Huang NM Radiman S Lim HN Khiew PS Chiu WS Lee KH Syahida A Hashim R Chia CH γ-Ray assisted synthesis of silver nanoparticles in chitosan solution and the antibacterial properties Chem. Eng. J 2009 155 499 507

[7] Darroudi M Ahmad MB Shameli K Abdullah AH Ibrahim NA Synthesis and characterization of UV-irradiated silver/montmorillonite nanocomposites Solid State Sci 2009 11 1621 1624

[8] Yin H Yamamoto T Wada Y Yanagida S Large-scale and size-controlled synthesis of silver nanoparticles under microwave irradiation Mater. Chem. Phys 2004 83 66 70

[9] Kim H-S Lee K-H Kim S-G Growth of monodisperse silver nanoparticles in polymer matrix by spray pyrolysis Aerosol. Sci. Tech 2006 40 536 544

[10] Lin C-A Tsai H-C Tung C-C Preparation of silver nanoparticles/pseudo-thermoplastic polyvinyl alcohol (PT-PVA) films by the synchronous chemical reduction method Polym.-Plast. Technol 2009 48 1171 1175

[11] Faraha AA Alvarez-Puebla RA Fenniri H Chemically stable silver nanoparticle-crosslinked polymer microspheres J. Colloid Interf. Sci 2008 319 572 576 10.1016/j.jcis.2007.11.030 18187145

[12] Zheng M Gu M Jin Y Jin G Optical properties of silver-dispersed PVP thin film Mater. Res. Bull 2001 36 853 859

[13] Luo C Zhang Y Zeng X Zeng Y Wang Y The role of poly(ethylene glycol) in the formation of silver nanoparticles J. Colloid Interf. Sci 2005 288 444 448 10.1016/j.jcis.2005.03.005 15927611

[14] Singh N Khanna PK *In situ* synthesis of silver nano-particles in polymethylmethacrylate Mater. Chem. Phys 2007 104 367 372

[15] Zhou Y Yu SH Wang CY Li XG Zhu YR Chen ZY A novel ultraviolet irradiation photo-reduction technique for preparation of single crystal Ag nanorods and Ag dendrites Adv. Mater 1999 11 850 852

[16] Khanna PK Singh N Charan S Viswanath AK Synthesis of Ag/polyaniline nanocomposite via an *in situ* photo-redox mechanism Mater. Chem. Phys 2005 92 214 219

[17] Zhang Z Zhang L Wang S Chen W Lei Y A convenient route to polyacrylonitrile/silver nanoparticle composite by simultaneous polymerization–reduction approach Polymer 2001 42 8315 8318

[18] Abu Bakar NHH Ismail J Abu Bakar M Synthesis and characterization of silver nanoparticles in natural rubber Mater. Chem. Phys 2007 104 276 283

[19] Huang NM Lim HN Radiman S Khiew PS Chiu WS Hashim R Chia CH Sucrose ester micellar-mediated synthesis of Ag nanoparticles and the antibacterial properties Colloid Surface A 2010 35 69 76

[20] Cai J Kimura S Wada M Kuga S Nanoporous cellulose as metal nanoparticles support Biomacromolecules 2009 10 87 94 19053296 10.1021/bm800919e

[21] Huang H Yuan Q Yang X Preparation and characterization of metal-chitosan nanocomposites Colloid Surface B 2004 39 31 37 10.1016/j.colsurfb.2004.08.014 15542337

[22] Raveendran P Fu J Wallen SL Completely “green” synthesis and stabilization of metal nanoparticles J. Am. Chem. Soc 2003 125 13940 13941 14611213 10.1021/ja029267j

[23] Singh M Sinha I Mandal RK Role of pH in the green synthesis of silver nanoparticles Mater. Lett 2009 63 425 427

[24] Singh M Sinha I Singh AK Mandal RK Formation of fractal aggregates during green synthesis of silver nanoparticles J. Nanopart. Res 2010 10.1007/s11051-010-0001-8

[25] Singh M Sinha I Premkumar M Singh AK Mandal RK Structural and surface plasmon behavior of Cu nanoparticles using different stabilizers Colloid Surface A 2010 359 88 94

[26] Xu X Zhou M Antimicrobial gelatin nanofibers containing silver nanoparticles Fiber. Polym 2008 9 685 690

[27] Bohren CF Huffman DR Absorption and Scattering of Light by Small Particles John Wiley & Sons Inc New York, NY, USA 1998

[28] Heath JR Size dependent surface plasmon resonances in bare silver particles Phys. Rev. B 1989 40 9982 9985 10.1103/physrevb.40.9982 9991532

[29] Aragon SR Elwenspoek M Mie scattering from thin spherical bubbles J. Chem. Phys 1982 77 3406 3413

